# Seroprevalence of SARS-CoV-2 in German secondary schools from October 2020 to July 2021: a longitudinal study

**DOI:** 10.1007/s15010-022-01824-9

**Published:** 2022-04-23

**Authors:** Carolin Kirsten, Elisabeth Kahre, Judith Blankenburg, Leonie Schumm, Luise Haag, Lukas Galow, Manja Unrath, Paula Czyborra, Josephine Schneider, Christian Lück, Alexander H. Dalpke, Reinhard Berner, Jakob Armann

**Affiliations:** 1 Department of Pediatrics, University Hospital and Medical Faculty Carl Gustav Carus, Technische Universität Dresden, Dresden, Germany; 2 Medical Faculty Carl Gustav Carus, Institute for Virology and Institute for Medical Microbiology and Hygiene, Technische Universität Dresden, Dresden, Germany; 3 University Children’s Hospital, Fetscherstrasse 74, 01307 Dresden, Germany

**Keywords:** Infectious disease, SARS-CoV-2, Seroprevalence, Pediatrics, Students, Teachers

## Abstract

**Purpose:**

To quantify the number of SARS-CoV-2 infections in students and teachers in 14 Secondary schools in eastern Saxony, Germany. Seroprevalence of SARS-CoV-2 antibodies in study population. Number of undetected cases.

**Methods:**

Serial seroprevalence study.

**Results:**

The role of educational settings in the SARS-CoV-2 Pandemic is still controversial. Seroprevalence increases from 0.8 to 5.9% from October to December when schools remained open and to 12.2% in March/April during a strict lockdown with closed schools. The ratio of undetected to detected cases decreased from 0.76 to 0.44 during the study period.

**Conclusion:**

During the second and third wave of the pandemic in Germany, students and teachers are not overrepresented in SARS-CoV-2 infections. The percentage of undetected cases is moderate and decreases over time. The risk of contracting SARS-CoV-2 within the household is higher than contracting it in educational settings making school closures rather ineffective in terms of pandemic control measures or individual risk reduction in children and adolescents.

**Trial registration:**

DRKS00022455 (July 23rd, 2020).

**Supplementary Information:**

The online version contains supplementary material available at 10.1007/s15010-022-01824-9.

## Introduction

Since the beginning of the SARS-CoV-2 pandemic [1], more than 195 countries implemented school closures as part of their pandemic control measures [2] hoping to effectively mitigate the spread of the virus within the whole population. Even in the second year of the pandemic data proving their effectiveness are largely missing [3] while numerous studies show that children are less likely to transmit SARS-CoV-2 compared to adults [4–6], tracing studies found only minimal transmission in educational settings [7–9] and surveillance data demonstrate minor increases in case numbers after schools re-opened [10–12]. Even the emerge of the Delta-Variant did not change these patterns [13].

However, even in countries with effective vaccination programs where widespread mitigation measures become less important with most of the vulnerable population being protected from severe disease courses, restrictions in educational settings partly remain. As a justification, these ongoing restrictions are now announced to address the individual risk of children and adolescents when contracting SARS-CoV-2.

While morbidity and mortality of the acute SARS-CoV-2 infection in children and adolescents is minimal [14], disease burden and severity of long-term post-COVID-19 complications are not yet reliably assessable [15, 16]. Given the clearly documented negative effects of school closures on the student population [17, 18], reliable data on the role of in-school SARS-CoV-2 transmission are urgently needed to inform and support public health officials in their decisions.

Seroprevalence samples of students and teachers at different time points during the second and third wave of the pandemic in Germany allow us to investigate the role of educational setting in the transmission of SARS-CoV-2 during different settings.

## Methods

### Study design

Students of grades 8–12 and their teachers in 14 secondary schools in Eastern Saxony were invited to participate in the SchoolCoviDD19 study. Schools were chosen by the state office for schools and education (Landesamt für Schule und Bildung—LASUB) without the involvement of the study team. At each school, all eligible students and teachers were invited to participate.

**Fig. 1:**
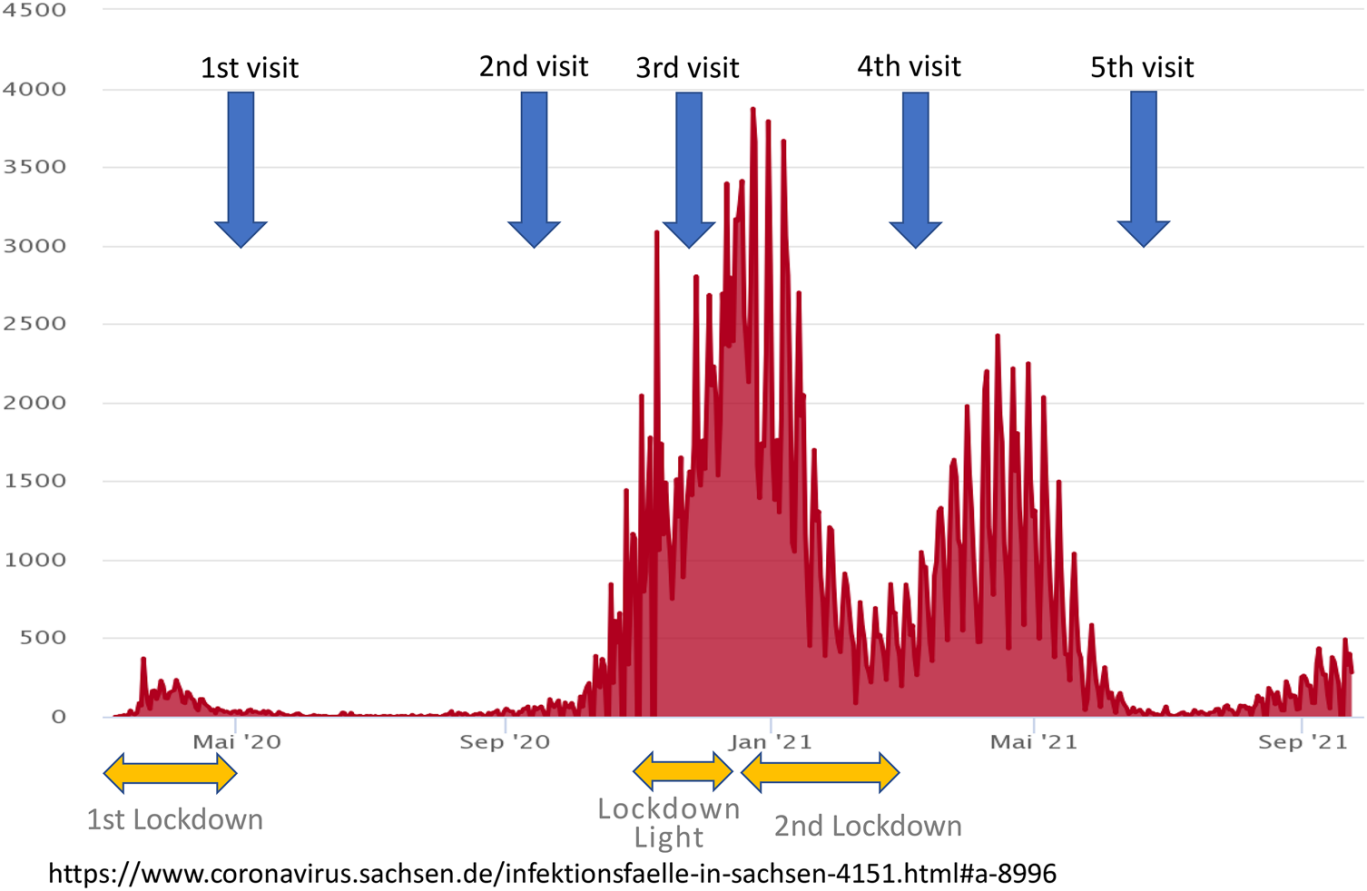
PCR-confirmed SARS-CoV-2 infections in Saxony between March 2020 and September 2021. Timepoints of study visits in the schools (blue arrows) and the timelines of lockdowns (yellow arrows)

The SchoolCoviDD19 study started with a first visit in May 2020 and participants were repeatedly sampled every 4–5 months. Results from the May and October sample are already published [19, 20]. Participation rates varied from 12 to 50% per school. After teachers, students and their legal guardians provided informed consent, 5 mL of peripheral venous blood was collected from each individual during visits at each participating school in March and April 2021 after their reopening on March 15th as well as in June and July 2021, another 3 months later (Fig. 1). In addition, participants were asked to complete a questionnaire on age, household size, previously diagnosed SARS-CoV-2 infections in themselves or their household contacts, officially mandated quarantine measures, respiratory symptoms, vaccination against SARS-CoV-2 and fear of an infection with SARS-CoV-2.

Household members of seropositive participants were invited to have their SARS-CoV-2 serostatus assessed via the FamilyCoviDD19 study. This study investigates transmission of SARS-CoV-2 in households with a questionnaire and the serostatus of each household member.

### Mitigation strategies

Students were not allowed to attend school if they were tested positive for SARS-CoV-2, had close contact to an infected individual within 14 days or showed symptoms of a respiratory infection—with the exception of an isolated runny or stuffed nose—until symptoms resolved for more than 48 h or tested negative for SARS-CoV-2.

Starting on November 1st, 2020, (“Lockdown light”) schools remained open with in-person teaching in full classes. Students were seated 1.5 m apart in classrooms, mask wearing in common areas was strongly recommended for students and teachers but not mandated. Student mixing was decreased by a reduction in extracurricular activities.

Starting December 12th, 2020, until March 14th, 2021, there was a strict lockdown with remote learning only, except for graduation classes who already went to school mid of January in split classes.

Starting March 15th, 2021, students returned to school in split classes with twice weekly self-testing and mandated mask wearing.

All of these measures were implemented by the Federal State of Saxony, they were not part of the study protocol nor assessed or controlled by the study team.

### Approval

The SchoolCoviDD19 study was approved by the ethics committee of the Technische Universität Dresden (BO-EK-156042020) and was registered on July 23rd, 2020, and assigned the clinical trial registration number DRKS00022455.

The FamilyCoviDD19 study was approved as well (BO-EK-342072020) and registered on September 7th, 2020 (DRKS00022564).

### Laboratory analysis

We assessed SARS-CoV-2 IgG antibodies in all samples using a commercially available chemiluminescence immunoassay technology for the quantitative determination of anti-S1 and anti-S2 specific IgG antibodies to SARS-CoV-2 (DiaSorin LIAISON SARS-CoV-2 S1/S2 IgG assay—sensitivity, 97.6%; specificity, 99.3%). For the detection of SARS-CoV-2-antibodies, serum was used. Antibody levels > 15 AU/mL were considered positive, and levels between 12 and 15 AU/mL were considered equivocal.

All samples with a positive or equivocal LIAISON test result, as well as all samples from participants with a reported personal or household history of a SARS-CoV-2 infection, were retested with two additional serological tests: these were a chemiluminescent microparticle immunoassay intended for the qualitative detection of IgG antibodies to the nucleocapsid protein of SARS-CoV-2 (Abbott Diagnostics ARCHITECT SARS-CoV-2 IgG—specificity, 99.6%; sensitivity, 97.9%) (an index (S/C) of < 1.4 was considered negative, whereas one ≥ 1.4 was considered positive) and an ELISA detecting IgG against the S1 domain of the SARS-CoV-2 spike protein (Euroimmun Anti-SARS-CoV-2 ELISA—specificity, 98.3%; sensitivity, 96.9%) (a ratio of < 0.8 was considered negative, 0.8–1.1 equivocal and > 1.1 positive).

Participants whose positive or equivocal LIAISON test result could be confirmed by a positive test result in at least one additional serological test were considered having antibodies against SARS-CoV-2.

### Statistical analysis

Analyses were performed using IBM SPSS V.25.0 and Microsoft Excel 2010. Results for continuous variables are presented as medians with IQR and categorical variables as numbers with percentages, unless stated otherwise.

A sample size calculation was performed based on an expected seroprevalence of 1% with 5% precision and a 95% confidence level, which yielded a minimum sample size of 500 participants.

## Results

### Study population/demographics

In March/April 2021, 1587 students and 457 teachers from 14 different schools—1741 participants in the city of Dresden, 271 in the county of Bautzen, 32 in the county of Görlitz—had their serostatus analyzed. 93 teachers and 7 students were excluded from the analysis because they were already vaccinated against SARS-CoV-2. Median age of the remaining students and teachers was 15 and 49, respectively; 55% of students and 71% of teachers were female (see Table 1 for full demographic data).

**Table 1:** Demographic data March/April 2021 and June/July 2021

	March/April 2021		June/July 2021	
All participants	2044		1609	
Vaccinated participants	100 (4.9%)		721 (44.8%)	
Unvaccinated Seropositive	238 (11.6%)		195 (12.1%)	
Unvaccinated Seronegative	1706 (83.5%)		692 (43.0%)	
Students^a^	1580		841	
Serostatus	Seropositive	Seronegative	Seropositive	Seronegative
*N*	203 (12.8%)	1377 (87.2%)	181 (21.5%)	660 (78.5%)
Median age (IQR)	15 (14–17)	15 (14–16)	15 (13–16)	14 (13–15)
Female sex (%)	112 (55)	767 (55)	96 (53)	368 (56)
Median household size (IQR)	4 (4–5)	4 (3–5)	4 (4–5)	4 (4–5)
Teachers^a^	364		46	
Serostatus	Seropositive	Seronegative	Seropositive	Seronegative
*N*	35 (10%)	329 (90%)	14 (30.4%)	32 (69.6%)
Median age (IQR)	47 (33.75–56)	50 (38–56)	45 (35–58.5)	46.5 (35–56.25)
Female sex (%)	22 (64)	239 (73)	10 (71)	21 (66)
Median household size (IQR)	3 (2–4)	2 (2–4)	2 (2–3)	2 (2–4)

*n* numbers, *IQR* Interquartile range

^a^Without vaccinated participants

238 participants—203 students and 35 teachers—had detectable antibodies against SARS-CoV-2 in at least two different assays and were therefore considered seropositive, indicating a seroprevalence of 12.2% overall—12.8% for students and 9.6% for teachers, respectively (*p* = 0.09 for the comparison), which is similar to a seroprevalence sample in children from May 2021 [19]. Seroprevalence in the participating schools in Dresden, however, was significantly lower than seroprevalence in the participating schools in in the county of Bautzen (10.9 vs. 22%; *p* < 0.01). While in Dresden there was no significant difference in the seroprevalence of students and teachers (11.2 vs 9.1%, NS), students in the county of Bautzen were more likely to be seropositive compared to their teachers (25 vs. 13.7%; *p* = 0.05) (Table 2).

**Table 2:** Seropositivity at study visit March/April 2021 and June/July 2021

March/April 2021								
		Participants^a^		Students		Teachers		*p*
		*n*/*N*	% (CI)	*n*/*N*	% (CI)	*n*/*N*	% (CI)	
All participants^a^	*n*/*N*	1944^b^		203/1580	12.8 (11.3–14.6)	35/364	9.6 (6.6.–12.6)	NS
Dresden	*n*/*N*	179/1644	10.9 (9.4–12.4)	154/1369	11.2 (9.6–12.9)	25/275	9.1 (5.8–12.7)	NS
Bautzen	*n*/*N*	59/269	21.9 (17.5–26.8)	49/196	25 (18.4–31.6)	10/73	13.7 (6.8–21.9)	0.05
*p*		< 0.01		< 0.01		NS		

June/July 2021								
		Participants^a^		Students		Teachers		*p*
		*n*/*N*	% (CI)	*n*/*N*	% (CI)	*n*/*N*	% (CI)	
All participants^a^	*n*/*N*	887^b^		181/841	21.5 (18.8–24.4)	14/46	30.4 (17.4–43.5)	NS
Dresden	*n*/*N*	146/724	20.2 (17.3–23.2)	134/670	20.0 (17.0–23.3)	12/54	22.2 (11.1–33.3)	NS
Bautzen	*n*/*N*	41/139	29.5 (21.6–36.7)	39/133	29.3 (21.8–37.6)	2/6	33.3 (0–66.7)	NS
*p*		0.018		0.02		NS		

*CI* Confidence interval, *n/N* numbers, *NS* not significant

^a^Not vaccinated

^b^Including 31 (March/April 2021)/24 (June/July 2021) participants of the region Görlitz in the East of Saxony

Seroprevalence at the beginning of the second wave in October 2020 in the same 14 schools was 0.8% (17/2091) indicating a 15-fold increase during the second wave of the pandemic [20, 21].

Analyzing only participants with available serostatus from both October 2020 and March/April 2021 (*n* = 1544) seroprevalence increased 13-fold from 0.9% (14/1544) to 11.5% (178/1544) (supplemental Table 1, supplemental Fig. 1).

In June/July 2021, 1234 students and 375 teachers participated in the study. 329 teachers (88%) and 392 (32%) students were already vaccinated at least once. Excluding the vaccinated participants, 195 participants—181 students and 14 teachers—had detectable antibodies in at least two different assays, indicating an overall seroprevalence of 21.5% for students and 30% for teachers (difference not significant), indicating a 1.7-fold increase from the sample in March/April to June/July 2021.

Analyzing only participants with available serostatus from both timepoints in March/April 2021 and June/July 2021 (*n* = 742) seroprevalence increased from 16.8% (125/742) to 20.2% (150/742), indicating a 1.2-fold increase (supplemental Table 1).

### Seroprevalence in schools in relation with overall case numbers

Cumulative laboratory PCR-confirmed cases per the statutory notification system in Dresden on April 1st, 2021, were 4501/100,000 increasing 28-fold from 160/100,000 on October 1st, 2020. Case numbers in the county of Bautzen increased 34-fold from 208/100,000 to 7133/100,000. In Dresden on June 1st, 2021, cumulative laboratory confirmed cases were 5469/100,000 increasing 1.2-fold from April 1st, 2021—during the same period confirmed cases in Bautzen increased 1.2-fold as well (Table 3).

**Table 3:** Cumulative laboratory confirmed SARS-CoV-2 cases per statuary notification system

	October 1st, 2020		April 1st, 2021		Fold increase	June 1st, 2021		Fold increase
	Number of cases	Cases/100,000	Number of cases	Cases/100,000		Number of cases	Cases/100,000	
Dresden	893	160	25,060	4501	28	30,449	5469	1.2
Bautzen	625	208	21,462	7133	34	27,261	9060	1.3

In December 2020, just one week before the strict lockdown on December 12th, 2020, 409 participants had their serostatus analyzed with 24 being seropositive indicating a seroprevalence of 5.9% at this timepoint. Additionally, time of infection in 107 seropositive cases between November 2020 and March 2021 could be determined exactly by date of PCR testing in a participant or household member. 52/107 (49%) of these infections took place during the “Lockdown light” until December 11th, 2020, with schools remaining open, while 55/107 (51%) happened during the strict lockdown starting December 12th, 2020, with closed schools.

Similarly, 39/238 (16%) of the seropositive participants in March/April 2021 had their serostatus analyzed at the end of December 2020 as well. 19/39 (49%) were already seropositive at this time in this subsample.

150/195 (77%) of the seropositive participants in June/July 2021 had already participated in March/April 2021. 125 (83%) were already seropositive at this timepoint.

### Undetected cases

Of the 238 seropositive participants in March/April 2021, 135 (57%) reported having been tested positive for SARS-CoV-2 previously by PCR (themselves or a household member) while 103 (43%) of them reported having no knowledge of a previous SARS-CoV-2 infection. The ratio of undetected to detected cases was therefore 0.76. The ratio of undetected to detected cases in June/July 2021 decreased to 0.44. There was neither a significant difference between the undetected to detected ratio between students and teachers nor between the city of Dresden and the county of Bautzen for both time points March/April and June/July 2021 (supplemental Table 2). Seropositive participants were significantly more likely to report common cold like symptoms between October 2020 and March 2021 (64 vs 38%; *p* < 0.01) but not between March and June 2021 (59 vs. 41%, NS); 75% (March/April 2021) and 76% (June/July 2021) of participants who reported symptoms were not seropositive. In March/April 2021 we could detect 116 participants with a positive PCR result for SARS-CoV-2 themselves, in June/July 2021 we detected 46 participants. 16% (March/April 2021), respectively 15% (June/July 2021) of them had no detectable antibodies (supplemental Table 3).

In March/April 2021, 507 participants (26%) reported to have been in an officially mandated quarantine at least once. 147 (29%) out of those quarantined were seropositive. The undetected to detected ratio in quarantined participants was 0.16. 399 of the quarantined participants in March/April 2021 had no positive SARS-CoV-2 PCR result and 86% of them had no detectable antibodies. In June/July 2021, 50 participants reported to have been in a quarantine without a positive SARS-CoV-2 PCR. 70% of them were seronegative.

### Household transmission

Household members of seropositive participants were invited to have their serostatus analyzed as well via our FamilyCoviDD19 study. Further transmission could be studied in 48 households of seropositive participants. The secondary attack rate (SAR) of the index cases with an unknown infection was only half as high compared to the SAR of the index cases with a known SARS-CoV-2 infection (SAR 0.25 vs. 0.49). 17/48 (35%) of the seropositive participants were the only seropositive household members with no transmission into the household at all.

### Preferred teaching model/fear of infection

Significantly more teachers would have preferred an earlier school opening (22 vs. 9%; *p* < 0.01), but also reported a greater fear of an infection with SARS-CoV-2 than the students (23 vs. 10%; *p* < 0.01). Less than 20% of all participants reported a preference for a remote learning system, with the majority opting for hybrid learning. Significantly more students than teachers preferred complete in-person instruction though (21 vs 14%; *p* < 0.01) (Table 4).

**Table 4:** Questionnaire: reopening of schools, teaching model, fear of infection March/April 2021

	Students (*n* = 1580) (%)	Teachers (*n* = 364) (%)	*p*
Time of school reopening			
Too early	768 (49)	143 (39)	< 0.01
Just right	644 (41)	116 (32)	< 0.01
Too late	149 (9)	81 (22)	< 0.01
Preferred teaching model			
In-person instruction	325 (21)	50 (14)	< 0.01
Hybrid learning	945 (60)	240 (66)	0.03
Remote learning	294 (19%)	63 (17%)	NS
Fear of an infection			
Yes/rather yes	589 (37%)	201 (55%)	< 0.01

*n* numbers, *NS* not significant

Statistical test applied for *p* values: Fisher’s exact test

While the fear of an infection decreased overall from March/April to June/July 2021 (41 vs. 23%; *p* < 0.01), it was significantly lower in non-vaccinated compared to vaccinated individuals (23 vs. 32%; *p* < 0.01).

### Vaccination

In June/July 2021, significant more teachers than students were vaccinated twice (80 vs. 13%; *p* < 0.01). Comparing Dresden and Bautzen, significant more participants were vaccinated in Dresden (59 vs. 44%; *p* < 0.01). 453 (99%) of the completely vaccinated participants had S-protein antibodies. 23 (3%) of the vaccinated participants were also positive for antibodies against N-protein indicating a prior infection with SARS-CoV-2.

42% (355/841) students not having been vaccinated until July 2021 would accept a vaccination, 37% are not sure about getting vaccinated and 20% would reject a vaccination.

## Discussion

The role of school settings in the transmission of SARS-CoV-2 remains controversial. Tracing studies done in the summer of 2020 could only detect minimal SARS-CoV-2 transmission in educational settings [7, 8] with the obvious caveat that mild or even asymptomatic cases could have been missed. By using antibody assessment we can measure transmissions more objectively and find a sharp increase in seroprevalence in students and teachers during the second wave of the pandemic in Germany after a stagnant phase during the low-prevalence period over the summer 2020 [20]. This increase clearly mirrors the trends in the general population and is in line with a previous study from Germany [22] The increase in seroprevalence in the school setting does not exceed the increase in cumulative reported infection in the statutory notification system in the same area which suggests that students and teachers are not overrepresented in the number of SARS-CoV-2 infections and that educational settings most likely do not act as drivers of the pandemic (Table 3).

While our study is one of the largest studies to report seroprevalence estimates in the student population in the later phase of the pandemic, the results are in line with a seroprevalence study in teachers showing no differences compared to a matched sample of blood donors [23] or to register data showing only minimal consequences for overall transmission if schools are kept open [12]. The regional differences in seroprevalence also support this finding. If educational setting were the primary location of transmission, regional differences should be less pronounced.

The fact that only half of the infections occurred during the “lockdown light” with schools remaining open while the other half occurred during the strict lockdown starting on December 11th, 2020, clearly shows that a relevant amount of children and adolescents contracts their infection in their household and that school closures will not be able to prevent these infections. Between March and June 2021 when schools remained open, the seroprevalence in schools increased not more than cumulative cases in the general population in Saxony (1.7 vs. 1.2-fold).

Given these facts and the clearly documented negative effects of school closures on the student population in terms of education [24], mental health [25], social contacts and control [17] as well as children’s nutrition [18], school closures seem unreasonable in most circumstances both as general pandemic control measure as well as an individual risk reduction in this age group.

In addition, we cannot find evidence of massive silent spread of SARS-CoV-2 infections. More than 50% of all seropositive participants knew of their infection and were quarantined accordingly, minimizing the risk of further spreading the virus. Moreover the ratio of undetected to detected cases did not increase during the second wave compared to the first wave of the pandemic in Germany [20] and was even lower than reported in previous studies [26] suggesting that local health departments were able to effectively accomplish their objectives even with high case numbers. The undetected to detected ratio even decreased during the third wave with opened schools.

The fact that undetected index cases were less likely to transmit the virus within their household compared to known and therefore more likely symptomatic cases is in line with household transmission [5, 27] and viral load studies that show that mildly or asymptomatic patients have lower viral loads [28] and are less likely to transmit SARS-CoV-2. It also suggests that symptom-based screening algorithms and mitigation measures are effective by identifying people being more infectious. This assumption is supported by our findings that significant more participants with symptoms were seropositive. Nevertheless, 40% of all participants reported having had a respiratory infection in the last months but 80% of them were not SARS-CoV-2 seropositive.

In June/July 2021, the pronounced increase of seroprevalence in teachers from 9.6 to 30% is due to the fact that most of the teachers were already vaccinated and excluded from analysis.

There are several limitations to our study. Mainly, we cannot provide information on eligible but nonparticipating students and teachers in the selected schools. Due to waning of antibodies, seropositivity could underestimate the number of infections. However, available data in children and adolescents suggests stable antibody titers even months after the infection in this age group [20, 29]. This is also supported by the fact that all participants with detectable S-antibodies in the initial study visit in May/June 2020 continued to have detectable antibodies in March/April 2021 (*n* = 17). Furthermore, the statutory notification system captures PCR-confirmed cases while we are using seroprevalence and we compare a specific age group in our study to overall cases in the official numbers. The comparison of these numbers can therefore only be used to estimate trends and not for an accurate analysis.

In addition, only 78% of our sample participated in October 2020 and March/April 2021 and 67% in March/April and June/July 2021, potentially leading to a certain degree of selection bias. However, analyzing only participants with available serostatus October and March did not change our results. With an increasing number of vaccinated participants in June/July, however, there are relevant differences in seroprevalence when comparing all unvaccinated participants to only those with samples from more than one timepoint making these data harder to interpret.

## Conclusion

During the second and third wave of the pandemic in Germany, students and teachers appear to be not overrepresented among people with SARS-CoV-2 infections. Dynamics in seroprevalence changes mirrors development of incidences in the general population. The percentage of undetected cases in March/April 2021 is moderate (0.76) and does not increase with higher case numbers during the second and third wave (0.44 in June/July 2021) due to mandatory testing in the schools. The risk of contracting SARS-CoV-2 within the household is higher than contracting it in educational settings making school closures rather ineffective in terms of pandemic control measures or individual risk reduction in children and adolescents.

## Supplementary Information

Below is the link to the electronic supplementary material.

Supplementary file1 (DOCX 15 KB)

Supplementary file2 (XLSX 48 KB)

Supplementary file3 (DOCX 17 KB)

Supplementary file4 (DOCX 14 KB)
