# Supplementary material for: Seroprevalence of SARS-CoV-2 in German secondary schools from October 2020 to July 2021: a longitudinal study
Source: Infection. 2022 Apr 23;50(6):1483–90. doi: 10.1007/s15010-022-01824-9 (PMC9034260; doi:10.1007/s15010-022-01824-9)
Supplement: Supplementary file 1 — Supplementary file1 (DOCX 15 KB) [file 15010_2022_1824_MOESM1_ESM.docx]

**Supplemental Table 3. Seronegative participants who reported a positive PCR test or were in quarantine**

|  | March/April 2021 | June/July 2021 |
| --- | --- | --- |
| Positive PCR test | 116 | 46 |
| seronegative | 18 (16%) | 7 (15%) |
| Quarantine | 399 | 50 |
| seronegative | 344 (86%) | 35 (70%) |
