# Supplementary material for: Seroprevalence of SARS-CoV-2 in German secondary schools from October 2020 to July 2021: a longitudinal study
Source: Infection. 2022 Apr 23;50(6):1483–90. doi: 10.1007/s15010-022-01824-9 (PMC9034260; doi:10.1007/s15010-022-01824-9)
Supplement: Supplementary file 3 — Supplementary file3 (DOCX 17 KB) [file 15010_2022_1824_MOESM3_ESM.docx]

**Supplemental Table 1. Seropositivity October 2020 to June/July 2021**

|  | **October 2020** | | | **December 2020** | | | **March April 2021** | | | **June July 2021** | | |
| --- | --- | --- | --- | --- | --- | --- | --- | --- | --- | --- | --- | --- |
|  | % | n/N | CI | % | n/N | CI | % | n/N | CI | % | n/N | CI |
| All participants* | 0.8 | 17/2091 | 0.4-1.2 | 5.9 | 24/409 | 3.7-8.1 | 12.2 | 238/1944 | 10.7-13.7 | 22 | 195/887 | 19.3-24.7 |
|  |  | | |  | | |  | | |  | | |
| All participants* with samples in October 2020 and March/April 2021 | 0.9 | 14/1544 | 0.5-1.4 |  | | | 11.5 | 178/1544 | 10.0-13.1 |  | | |
|  |  | | |  | | |  | | |  | | |
| All participants* with samples in December 2020 and March/April 2021 |  | | | 6.4 | 20/311 | 3.4-9.0 | 12.5 | 39/311 | 9.3-16.4 |  | | |
|  |  | | |  | | |  | | |  | | |
| All participants* with samples in March/April and June/July 2021 |  | | |  | | | 17 | 126/742 | 14.2-19.7 | 20 | 150/742 | 17.4-23.4 |
| Students |  | | |  | | | 16.9 | 119/706 | 14.2-19.7 | 19.5 | 138/706 | 16.7-22.5 |
| Teachers |  | | |  | | | 19.4 | 7/36 | 8.3-33.3 | 33.3 | 12/36 | 19.4-50.0 |

*not vaccinated; CI *Confidence Interval;* n/N *numbers*
