# Supplementary material for: Seroprevalence of SARS-CoV-2 in German secondary schools from October 2020 to July 2021: a longitudinal study
Source: Infection. 2022 Apr 23;50(6):1483–90. doi: 10.1007/s15010-022-01824-9 (PMC9034260; doi:10.1007/s15010-022-01824-9)
Supplement: Supplementary file 4 — Supplementary file4 (DOCX 14 KB) [file 15010_2022_1824_MOESM4_ESM.docx]

**Supplemental Table 2. Undetected to detected ratio March/April 2021 and June/July 2021**

| **March/April 2021** | | | | |
| --- | --- | --- | --- | --- |
|  | **Detected cases*** | **Undetected cases** | **ratio** | **p** |
| Seropositive participants | 135 | 103 | 0.76 |  |
| Seropositive students | 113 | 90 | 0.8 | NS |
| Seropositive teachers | 22 | 13 | 0.59 |  |
| Seropositive participants in Dresden | 105 | 74 | 0.71 | NS |
| Seropositive participants in Bautzen | 30 | 29 | 0.97 |  |

| **June/July 2021** | | | | |
| --- | --- | --- | --- | --- |
|  | **Detected cases*** | **Undetected cases** | **ratio** | **p** |
| Seropositive participants | 135 | 60 | 0.44 |  |
| Seropositive students | 127 | 54 | 0.43 | NS |
| Seropositive teachers | 8 | 6 | 0.75 |  |
| Seropositive participants in Dresden | 102 | 42 | 0.4 | NS |
| Seropositive participants in Bautzen | 24 | 17 | 0.71 |  |

*participants tested positive by PCR or with a household member tested positive by PCR;

NS *not significant*
